# Retinal involvement and ocular findings in COVID-19 pneumonia patients

**DOI:** 10.1038/s41598-020-74446-6

**Published:** 2020-10-15

**Authors:** Maria Pia Pirraglia, Giancarlo Ceccarelli, Alberto Cerini, Giacomo Visioli, Gabriella d’Ettorre, Claudio M. Mastroianni, Francesco Pugliese, Alessandro Lambiase, Magda Gharbiya

**Affiliations:** 1 Department of Sense Organs, Sapienza University of Rome, Viale del Policlinico 155, 00161 Rome, Italy; 2 Department of Public Health and Infectious Diseases, Sapienza University of Rome, Rome, Italy; 3 Azienda Ospedaliero-Universitaria Policlinico Umberto I, Rome, Italy; 4 Department of Anaesthesia and Intensive Care Medicine, Sapienza University of Rome, Rome, Italy

**Keywords:** Eye manifestations, Viral infection

## Abstract

Changes in immune and coagulation systems and possible viral spread through the blood–brain barrier have been described in SARS-CoV-2 infection. In this study, we evaluated the possible retinal involvement and ocular findings in severe COVID-19 pneumonia patients. A cross-sectional study was conducted on 46 patients affected by severe COVID-19 who were hospitalized in one intensive care unit (ICU) and in two infectious disease wards, including bedside eye screening, corneal sensitivity assessment and retinography. A total of 43 SARS-CoV-2-positive pneumonia patients affected with COVID-19 pneumonia were included, including 25 males and 18 females, with a median age of 70 years [IQR 59–78]. Except for one patient with unilateral posterior chorioretinitis of opportunistic origin, of whom aqueous tap was negative for SARS-CoV-2, no further retinal manifestation related to COVID-19 infection was found in our cohort. We found 3 patients (7%) with bilateral conjunctivitis in whom PCR analysis on conjunctival swabs provided negative results for SARS-CoV-2. No alterations in corneal sensitivity were found. We demonstrated the absence of retinal involvement in SARS-CoV-2 pneumonia patients. Ophthalmologic evaluation in COVID-19, particularly in patients hospitalized in an ICU setting, may be useful to reveal systemic co-infections by opportunistic pathogens.

## Introduction

As a result of its pandemic spread and the very limited therapeutic options, COronaVIrus Disease 19 (COVID-19) is considered an unprecedented global health challenge. Italy was the first European country affected by a severe outbreak of the SARS-CoV-2 epidemic that emerged from the Wuhan region (China) and currently has 241.611 total cases and 34.861 deaths^1–3^. As a life-threatening condition, most of the research has primarily focused on the respiratory system for survival rate improvement, leaving the effects on other systems or districts still unclear or unknown^4–7^. Regarding eye involvement in COVID-19, only few data are currently available^8^. The subfamily of *Orthocoronavirinae*—in which SARS-CoVs belong to the *Betacoronavirus* genus*—*is already known to occasionally affect ocular structures^9–12^. Several manifestations, such as uveitis, retinitis and optic neuritis, have been described in animal models of murine and feline species; however, these findings have never been confirmed in humans^13^. The first evidence on humans came from a few studies conducted during the early 2000s severe acute respiratory syndrome (SARS) outbreak caused by SARS-CoV-1. SARS RNA was detected in the tears of small cohorts of patients, suggesting the eye as an entrance window for infection and/or as a hypothetical source of viral spread^14–16^. In more recent times, the SARS-CoV-2 epidemic has revived interest in ocular manifestations, especially since associated conjunctivitis has been described and colonization of the ocular surface has been reported. The same unsolved questions emerged in the 2000s, suggesting the implementation of quick precautionary strategies to protect ophthalmologists and their patients^17–21^. Nevertheless, although recent data suggest that COVID-19 infection may be associated with changes in immune and coagulation systems and possible viral spread through the blood–brain barrier, with clinical and anatomopathological findings of disseminated intravascular coagulopathy (DIC), the effects of these alterations on the eye, specifically regarding posterior segment involvement, have not been fully elucidated^13,22–24^.

The main objective of the present cross-sectional study was to explore the possible retinal involvement in COVID-19 and to provide data on ocular findings from SARS-CoV-2-positive pneumonia patients.

## Methods

### Setting

We conducted a prospective cross-sectional study at the *Policlinico Umberto I*, a large teaching hospital in Rome (Italy), that included a cohort of patients affected by COVID-19 who were hospitalized in one intensive care unit (ICU) and in two infectious disease wards from April 24 to May 24, 2020. The study and data accumulation were in conformity with the Italian laws. The research protocol was approved by the ethical board of the Sapienza University of Rome (Rif. 5965, Prot. 109/2020) and was conducted in accordance with the tenets of the Declaration of Helsinki. All patients gave written informed consent to the study protocol, including clinical data and biological sample collection.

During the study period, 68 patients were hospitalized at any stage of the disease in the abovementioned units. Twenty-one patients were not able to be screened because of continuous positive airway pressure (CPAP) therapy, and one patient denied consent. Therefore, we finally screened a total of 92 eyes of 46 patients. Patients were treated with ad interim best available therapy (BAT) according to the Italian Society of Infectious and Tropical Diseases (SIMIT): hydroxychloroquine 200 mg bid and azithromycin 500 mg daily plus tocilizumab 8 mg/kg (up to a maximum of 800 mg per dose) twice with an interval of 12 h. All patients were on weight-based low-molecular-weight heparin and systemic steroid treatment 0.5–1 mg/kg^25^. The inclusion criteria were as follows: (1) written informed consent signed, (2) age between 18 and 90 years, (3) confirmed positive results for SARS-CoV-2 from nasopharyngeal or oropharyngeal swab testing at the time of the ophthalmological assessment, and (4) lung involvement related to COVID-19. We excluded patients who had (1) active neoplasia or (2) a history of any ocular diseases such as glaucoma, uveitis, retinal vascular occlusion or major eye surgery performed within the previous six months. Based on the clinical conditions, patients were stratified following the COVID-19 phenotypic classification proposed by the Italian Society of Anesthesiology, Analgesia, Resuscitation and Intensive Care (SIAARTI): paucisymptomatic disease (Stage I), mild pneumonia (Stage II), moderate to severe pneumonia (Stage III), acute respiratory distress syndrome (ARDS, Stage IV), sepsis (Stage V), and septic shock (Stage VI)^26^. To classify anamnestic and prognostic comorbidities at baseline, we used the Charlson Comorbidity Index (CCI)^27^. Finally, at the time of ophthalmological screening, to assess the patient inflammatory status and thrombotic risk, we recorded the laboratory tests of the day. Erythrocyte sedimentation rate (ESR), C-reactive protein (CRP), white blood cell (WBC) count and lymphocyte (LYM) values were collected to evaluate the subject’s inflammatory status, while thrombotic risk was scored using the International Society on Thrombosis and Haemostasis (ISTH) criteria for DIC^28^.

### Virologic test

Oropharyngeal and nasopharyngeal swabs for diagnosis of COVID-19 were performed in duplicate for SARS-CoV-2 E-, S-, N-, RdRp- and RdRp/N- genes by a standardized reverse transcriptase polymerase chain reaction (RT-PCR) routinely used for diagnostic purposes. RT-PCR was run immediately after sample collection and results were available on the same day. The search for SARS-CoV-2 on biological samples coming from the eye was carried out with the same laboratory method.

### Ophthalmological evaluation

Bedside ophthalmologic evaluation was performed in both eyes and included ocular annexes and anterior segment examination using direct lighting and a 20-dioptre lens for magnification. In those cases, presenting unilateral or bilateral conjunctival hyperaemia, a conjunctival swab was performed in both eyes and a concomitant nasopharyngeal swab was repeated for RNA SARS-CoV-2 detection. Quantitative corneal sensitivity, scored from 0 to 6 (0 corresponding to the absence of sensitivity and 6 to the highest sensitivity), was further assessed following a previously described protocol, only in awake patients, using the Cochet-Bonnet aesthesiometer (COBO)^29^. Ocular fundus examination was performed after pharmacological pupil dilation with 1% tropicamide using binocular indirect ophthalmoscopy and a 20-dioptre lens. Images of the posterior pole were acquired by the same investigator (MPP) using a handheld fundus camera with a 40-degree field of view, 9 internal fixation targets for peripheral imaging and 5-megapixel resolution (Smartscope from Optomed, Oulu, Finland).

### Data source and collection

For every patient included in the study, we collected demographic data, systemic and ocular history, laboratory test results, medical administration data, and ocular findings. All data were recorded using an *electronic case report form* (eCRF) by one investigator (GV). Data are then securely transferred to a central database, where missing data or every discrepancy was corrected by a double-check analysis or after a collegial evaluation.

The primary outcome of the study was the rate of retinal involvement in SARS-CoV-2 -positive pneumonia patients. Secondary outcome was the rate of anterior segment findings, mainly conjunctival changes and corneal sensitivity alterations.

### Statistical analysis

Considering the relatively small sample size, normal distribution of data was analysed by the Shapiro–Wilk test. Continuous variables were reported as the mean, median, maximum and minimum values and interquartile ranges (IQR 25% and 75%). Categorical variables were reported as counts and percentages. *P* values of < 0.05 were considered as statistically significant. All analyses were performed using SPSS v. 25.0 (SPSS, Inc., Chicago, IL, USA).

## Results

### Characteristics of the cohort

Starting from 46 screened patients, based on the inclusion and exclusion criteria, we excluded two patients with active neoplasia and one with chronic glaucoma. Finally, a total of 43 subjects (25 males and 18 females) with a median age of 70 [IQR 59–78] were included in the present study. The patients were hospitalized after a median of 4 days (range, 0 to 11 days) from COVID-19 symptom onset, and ophthalmological screening was performed after a median of 21.5 days (range, 1 to 47 days) from hospitalization. The comorbidity index was calculated at admission and ranged from 0 to 7, with a median of 1. Ten out of 43 patients (9 male and 1 female) were screened in an ICU setting. The baseline anamnestic and clinical characteristics of the study cohort are shown in Table 1. Patients’ clinical status, as assessed by the SIAARTI COVID-19 classification, varied from stage II to stage V, with men globally in worse conditions than women. The COVID-19 stage distributions are shown in Table 2. Laboratory tests showed signs of systemic inflammation with general high CRP and low lymphocyte counts. Overall, D-dimer values were significantly elevated; however, only one patient (2.3%) had suggestive criteria for DIC according to ISTH, and 4 patients had a prior diagnosis of COVID-19-related pulmonary thromboembolism. Table 3 shows the principal laboratory test results in our cohort.

**Table 1:** Baseline anamnestic and clinical characteristics of the 43 patients enrolled.

Patients	n. 43 (%)	Median [IQR]
Sex (male)	25 (58.1%)	
Age		70 [59–78]
Male age		67 [60–76]
Female age		74 [57–85]
Days before hospitalization*		4.0 [3–6.5]
Days before screening**		21.5 [10–34]
CCI		1 [0–2]
Hypertension	22 (51.2%)	
Diabetes mellitus	8 (18.6%)	
CAD	7 (16.3%)	
COPD	7 (16.3)	
CVA or TIA	6 (14.0%)	
PAD	5 (11.6%)	
CHF	4 (9.3%)	
AF	4 (9.3%)	
Dementia	4 (9.3%)	
Hemiplegia	2 (4.7%)	
Liver disease	2 (4.7%)	

CCI, Charlson Comorbidity Index; CAD, Coronary Artery Disease; COPD, Chronic Obstructive Pulmonary Disease; CVA, Cerebrovascular Accident; TIA, Transient Ischemic Attack; PAD, Peripheral Artery Disease; CHF, Congestive Heart Failure; AF, Atrial Fibrillation.

*Days between symptoms onset and hospitalization.

**Days between hospitalization and first ocular screening.

**Table 2:** Cohort stratification using SIAARTI COVID-19 classification: paucisymptomatic disease (Stage I), mild pneumonia (Stage II), moderate to severe pneumonia (Stage III), acute respiratory distress syndrome (ARDS, Stage IV), sepsis (Stage V), septic shock (Stage VI).

COVID-19 stage	Total n. 43 (%)	Male n. 25 (%)	Female n. 18 (%)
I	0 (0%)	0 (0%)	0 (0%)
II	19 (44.2%)	9 (36%)	10 (55.6%)
III	9 (20.9%)	5 (20%)	4 (22.2%)
IV	11 (25.6%)	7 (28%)	4 (22.2%)
V	4 (9.3%)	4 (16%)	0 (0%)
VI	0 (0%)	0 (0%)	0 (0%)

**Table 3:** Overall inflammatory and coagulation status at the moment of ocular screening.

Lab tests	Median [IQR]
WBC (× 10^3^/µL)	5.7 [4.1–6.8]
LYM (× 10^3^/μL)	0.720 [0.51–1.06]
PLT (× 10^3^/µL)	228 [172.5–270]
INR	1.0 [1.0–1.1]
PTT (s)	28.6 [26.8–34.4]
Fibrinogen (mg/dL)	3.6 [2.7–5.6]
D-dimer (µg/L)	820.0 [389–1570]
PCR (mg/L)	4440 [550–22150]
ESR (mm/h)	33.0 [13–51.5]

Only one patient had suggestive criteria for Disseminated Intravascular Coagulation.

WBC, White Blood Cells; LYM, Lymphocytes; PLTs, Platelets; INR, International Normalized Ratio; PTT, Partial Thromboplastin Time; CRP, C-Reactive Protein; ESR, Erythrocyte sedimentation rate.

### Ocular posterior segment findings

**Figure 1:**
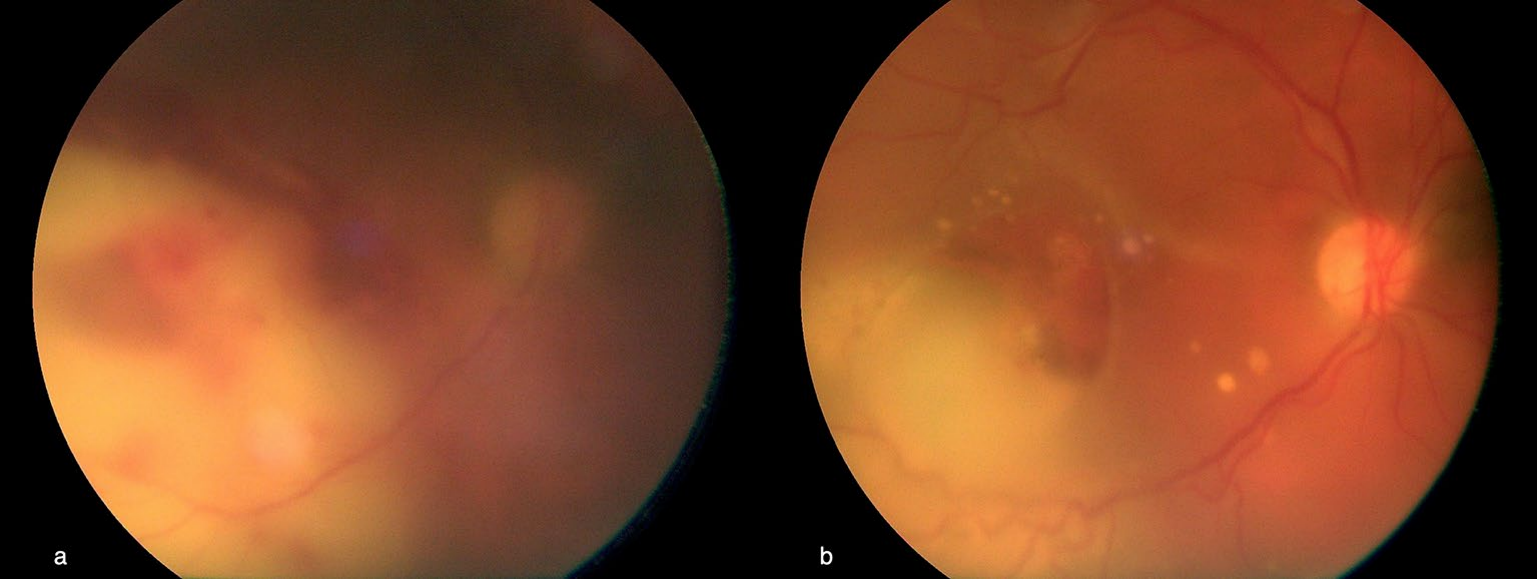
Fundus image of the patient with unilateral chorioretinitis at the time of first eye examination (**a**) showing an area of deep chorioretinal exudation involving the posterior pole associated with deep retinal haemorrhages. Fundus image of the same eye 3 weeks after IV amphotericin B, the extent of the chorioretinal lesion is reduced, with sharper margins and pigment mottling at both the subretinal and sub-RPE level (**b**).

Apart from one patient with unilateral posterior chorioretinitis not due to SARS-CoV-2 that is discussed below, no further retinal manifestation related to COVID-19 infection was found in our cohort (rate of COVID-19 related retinal manifestation: 0/43 patients, 0%). The patient with chorioretinitis, was a 67-year-old male, hospitalized in the ICU for stage V disease, who presented grade 1 vitreous haze, and a wide area of deep chorioretinal whitening involving the posterior pole, associated with deep retinal haemorrhages (Fig. 1a). According to the standard protocol, an aqueous tap to rule out possible pathogens, including SARS-CoV-2, was performed. A diagnosis of probable fungal retinitis was made, and the systemic antifungal therapy was changed accordingly, by replacing IV caspofungin with amphotericin B. Microbiological tests excluded the presence of SARS-CoV-2 in aqueous humour, and blood culture subsequently confirmed the diagnosis of *Candida parapsilosis* infection. Chorioretinitis gradually improved (Fig. 1b), and blood culture became sterile, however, the patient died of SARS-CoV-2 -related pneumonia 4 weeks later.

### Ocular anterior segment findings

We observed 3 cases (7%) of bilateral conjunctivitis characterized by mild serous secretion, both bulbar and tarsal conjunctival hyperemia with no chemosis: two patients were in stage II and one was in stage III of the SIAARTI COVID-19 classification. In all three patients, the PCR assay from conjunctival swabs for the detection of SARS-CoV-2 provided negative results while the virus RNA was still present from nasopharyngeal swab testing. The corneal sensitivity score in screened eyes, as assessed by esthesiometry, ranged from 4 to 6 with a median of 5. Table 4 shows the ocular findings observed in our cohort.

**Table 4:** Anterior and posterior segments findings in the 43 enrolled patients.

Ocular findings	n (%)
Hypertensive retinopathy	4 (9.3%)
Conjunctival hyperemia	3 (7%)
Age-related Macular degeneration	2 (4.6%)
Diabetic retinopathy	1 (2.3%)
Chorioretinitis	1 (2.3%)

## Discussion

The primary outcome of the study was to evaluate the presence of posterior segment alterations through a cross-sectional sample at different stages of the COVID-19, and to the best of our knowledge, this is the first study to address this issue in a cohort of hospitalized people positive for SARS-CoV-2. All available evidence in humans is focused on the ocular surface, where almost only conjunctivitis has been described, and most data come from case reports or findings from human conjunctival samples^17,30,31^. In our patients, of whom we carefully considered baseline anamnestic, the results of fundus examination seem to demonstrate that neither the retina nor retinal vessels are involved in the active phase of COVID-19 infection.

The rationale for focusing on potential ocular fundus alterations relies on previous studies conducted in animals infected by viruses belonging to the large subfamily of *Orthocoronavirinae.* The occasional onset of uveitis or retinitis in feline and murine models has been described and linked to an underlying autoimmune process inducing vasculitis or to viral-mediated inflammation^9–13^. Recent clinical and anatomopathological reports have described endothelial damage as one of the most prominent causes of the systemic vascular thromboembolic and/or inflammatory manifestations of COVID-19^32–34^. In this setting, the retina, as a privileged district for noninvasive and in vivo evaluation of systemic diseases, may reveal alterations such as vascular occlusion related to thrombotic susceptibility and chorioretinitis or vasculitis directly mediated by the virus. Hence, as reported in the brain, we considered the possibility of a direct ocular spread of SARS-CoV-2 through the two blood-retinal barriers (BRBs). In the recent literature on COVID-19, there are only anecdotal reports of virus spread through the blood–brain barrier^35–37^. Although two proven cases of positive CSF testing for SARS-CoV-2 have been described and one post-mortem, there are no certain data proving that the virus is able to directly affect the central nervous system^38,39^. Our findings, on a cohort of subjects in different stages of the disease, including ICU patients, seem to demonstrate that SARS-CoV-2 may not be able to cross ocular BRBs. Furthermore, to the best of our knowledge, we report the first attempt to isolate SARS CoV-2 in human aqueous humour.

With respect to the known thrombotic susceptibility described in COVID-19, we did not find any sign of retinal vascular involvement, such as venous or arterial occlusion. However, it should be considered that all patients in our cohort were treated with low-molecular-weight heparin to prevent systemic vascular complications, and only one patient addressed the criteria for DIC.

At the present time, there is only one report that described retinal lesions in a small cohort of asymptomatic SARS-CoV-2-positive patients. The authors found cotton-wool-like lesions and microhaemorrhages in 4 out of 12 patients and inner retinal OCT hyperreflective spots in the whole sample. However, apart from “normal blood parameters”, the authors did not provide any specific information enabling the clinical characterization of their patients. Indeed, no data regarding the presence of systemic comorbidities as well as no details regarding the patients’ ongoing therapy were given. Hence, it cannot be excluded that their findings may be ascribed to pre-existing non-COVID-19-related systemic diseases affecting the retina, such as hypertensive or diabetic retinopathy or other infectious diseases^40–43^. In our cohort, largely composed of subjects with severe COVID-19, we merged retinal findings with baseline anamnestic findings. Nevertheless, our negative results should also be attributed to the ongoing immune modulating treatment, including steroids or tocilizumab, that could have concealed ophthalmoscopic findings. Finally, with regard to patients with chorioretinitis from fungal sepsis, it should be considered that severe COVID-19 patients, especially when hospitalized in an ICU setting, may be affected by superinfection due to several opportunistic pathogens with possible eye localization^44^.

Regarding the anterior segment findings, the literature describes conjunctivitis as a part of the clinical manifestation of COVID-19 with a variable rate of presentation, ranging from 0 to 32%. Only in 4–7% of cases did PCR reveal the presence of SARS-CoV-2 from conjunctival swabs^17,19,31,45^. In all three patients presenting bilateral conjunctivitis that was observed in our study, the conjunctival swab was negative for SARS-CoV-2. Nevertheless, since other viruses (e.g., herpes and adenovirus) are known to induce and have been detected in conjunctivitis, we cannot exclude the transient presence of SARS-CoV-2 on the ocular surface at any time before or after our swab^46^. Alternatively, conjunctivitis may represent an epiphenomenon in hospitalized patients. Since it has been definitely demonstrated that COVID-19 affects the peripheral nervous system, as shown by the reported 85–88% rate of olfactory and gustatory dysfunction, we investigated the possible involvement of trigeminal sensory pathways by exploring corneal sensitivity^47^. However, the results of aesthesiometry seem to demonstrate that, unlike herpes viruses, SARS-CoV-2 does not affect corneal sensitivity^48^.

One strength of this cross-sectional study is to have explored posterior segment involvement in COVID-19 pneumonia patients at different stages of disease and in patients who had different comorbidities at the time of their hospitalization. Furthermore, considering our brief study period and the actual feasibility of an ophthalmological evaluation in a bedside setting, we obtained data from a relevant sample size even if a larger study population is probably necessary to confirm our findings. One important limitation of the present study is the unavailability of genomic characterization and phylogenetic analysis of SARS‐COV‐2 involved in the infections of our cohort. Among the other shortcomings, potential bias resulting from systemic therapies put in place before the ophthalmological evaluation and those deriving from the exclusion of patients in CPAP therapy should be disclosed. Finally, we cannot exclude the presence of COVID-19 related subclinical retinal alterations. Hence, future studies investigating retinal involvement in COVID-19 patients should consider the use of diagnostic tools, such as optical coherence tomography or fluorescein angiography, to confirm our findings.

## Conclusions

In conclusion, our study demonstrated the absence of retinal manifestations in SARS-CoV-2 pneumonia patients. Given the frequent drug-induced immunological dysfunction, ophthalmological evaluation may be useful to reveal systemic coinfections by opportunistic pathogens, especially in ICU patients.

### Ethical approval

Ethical approval was obtained from the Ethics Committee of Policlinico Umberto I (approval number/ID Prot. 109/2020).
